# 

**Published:** 1888-12

**Authors:** 


RECEIPTED—1888—.Drs. J. B. Price, Z. T. Young, A. H. Redding; J. C. Kerr, T. M. Battey, J. M. McMichal, W. A. Dobbs, F. H. Davis, L. L. Clement.

1889.—Drs. J. S. Horseley, C. S. Merrick, L. H. Hill. to July; J. H. Henry, to July; R. L. Moody, James Nesbit, K. L. Thornton, Wm. Meeks, J. W. Nelms.

1887.—T. J. Brasher, Robt. Phelps, R. R. Salter, T. L. Christian, M. E. Meggs.

